# *In Vivo* investigation of xenotransplanted human blood-derived scaffold into mice as a biodegradable construct for improvement of pelvic floor repair

**DOI:** 10.3389/fbioe.2025.1627538

**Published:** 2025-09-26

**Authors:** Fariba Behnia-Willison, Pouria Aryan, Mojdeh Salehnia, Nadia Willison, Tran T. T. Nguyen, Nelson Tansu, Derek Abbott

**Affiliations:** ^1^ FBW Gynaecology Plus, Ashford, SA, Australia; ^2^ Discipline of Biomedical Engineering, School of EME, The University of Adelaide, Adelaide, SA, Australia; ^3^ Anatomy Department, Faculty of Medical Sciences, Tarbiat Modares University, Tehran, Iran

**Keywords:** autologous, blood-derived, scaffold, biodegradation, biocompatibility

## Abstract

**Objective:**

We developed an autologous, novel human blood-derived scaffold (hBDS) to improve pelvic floor disorders. Both *in vivo* biocompatibility and biodegradation were investigated and compared by subcutaneous implantation, in the abdominal wall, and the back muscle of mice.

**Methods and Materials:**

After preparing the scaffold, it was xenotransplanted subcutaneously, and *in vivo* biodegradation and host responses were assessed morphologically using hematoxylin, eosin, and Masson’s trichrome staining over 6 weeks. Immunohistochemistry for the CD136 marker was conducted to evaluate vascularization. In another series of experiments, the scaffold was sutured at multiple points on the abdominal wall and back muscle to prolong its biodegradation time and assess the scar formation around the transplantation site.

**Results:**

Three days after implantation, no infection or severe inflammation was observed, and the scaffold was surrounded by connective tissue and fibroblasts, indicating initial scaffold degradation. By 1 week, the scaffold exhibited high biodegradation and increased fibroblast infiltration. Scaffold degradation was extensive at 2 weeks, with continued fibroblast infiltration and new collagen deposition. By 3 weeks, the scaffold had completely degraded, with minimal inflammation. The normal dermal structure was restored by weeks four to six after transplantation. Immunohistochemistry confirmed the neovascularization at one- and 2-week post- transplantation. Suturing the scaffold on the peritoneum and back muscle resulted in higher fibroblast infiltration and collagen formation around the suture compared to the non-suture group, with no morphological differences between the abdominal wall and back muscle reactions.

**Conclusion:**

The novel human blood-derived scaffold demonstrated biodegradation and high biocompatibility. Suturing the scaffold on the abdominal wall or back muscle effectively improves clinical symptoms, while further improvements are needed for its clinical application.

## Introduction

Pelvic floor disorders (PFDs) are common conditions affecting a significant portion of the population, particularly women, and affect their lifestyle. Recent literature indicates that approximately one in four women in the United States experiences at least one type of PFD, such as urinary incontinence, fecal incontinence, or pelvic organ prolapse (Martínez‐Galiano et al., 2024; Peinado Molina et al., 2023; Palmieri et al., 2022). The prevalence of these disorders increases with age, with more than 50% of women over the age of 80 affected. Childbirth, aging, obesity, hormonal changes that are associated with menopause, previous pelvic surgeries, and some pulmonary diseases that cause chronic coughing or straining are the main etiological factors of PFDs (Martínez‐Galiano et al., 2024; Peinado Molina et al., 2023; Palmieri et al., 2022; Peinado-Molina et al., 2024; Torosis et al., 2024).

For the treatment of the PFD, there are both non-surgical and surgical methods (Torosis et al., 2024; Quaghebeur et al., 2021). In non- surgical treatment, the manual, stimulation, or relaxation technique is applied (Quaghebeur et al., 2021). Despite the effectiveness of the surgical treatment by synthetic mesh implantation, it resulted in complications such as mesh erosion and chronic pain. Because the synthetic mesh is composed of non-degradable microporous monofilament fibers. Therefore, it is required for new therapeutic strategies (Powers et al., 2019; Seifalian et al., 2023).

Tissue engineering represents a promising frontier in the treatment of PFDs, offering innovative solutions to restore the function of the pelvic floor by regenerating muscle, connective tissue, and neural structures (Lin et al., 2022; Wu et al., 2020). Different types of biomaterials have been used in tissue engineering that mimic the extracellular matrix in living tissues. Among them, the natural scaffold serves as a supportive structure for cell attachment, proliferation, and differentiation into functional tissues. Natural scaffolds offer several advantages that make them suitable for various medical applications. As they are derived from biological sources such as collagen, fibrin, hyaluronic acid, and decellularized tissues, they are biocompatible. Furthermore, natural scaffolds contain bioactive molecules, growth factors, and cytokines, that involve and enhance tissue regeneration (Wu et al., 2020; Brownell et al., 2022). Autologous natural scaffolds are obtained from the individual own tissues, offering specific advantages such as their immunocompatibility or biocompatibility, which minimizes the risk of rejection or adverse immune responses and facilitates personalized treatment approaches (Wu et al., 2020; Brownell et al., 2022). Despite challenges such as limited availability and variability in quality, ongoing research aims to overcome these limitations and further advance the field of regenerative medicine (Henderson et al., 2024; Chapple et al., 2015; Yuan et al., 2021). It seems that blood-derived materials may be a suitable alternative to reduce these challenges (Henderson et al., 2024; Prodromidou et al., 2022a; Dankova et al., 2023). Recent studies have shown that the loaded scaffolds, with cells harvested from the patient or stem cells, can facilitate the regeneration of pelvic floor tissues (Wu et al., 2020; Manodoro et al., 2022; Wen et al., 2013). Mesenchymal stem cells with bone marrow, adipose tissue, endometrial and urine sources, embryonic stem cells, muscle tissue stem cells, and induced pluripotent stem cells have shown great promise due to their ability to differentiate into various cell types (Manodoro et al., 2022). Recently, an experimental study evaluated the regenerative potential of bone marrow-derived mesenchymal stem cells in the repair of the rat model of an injured vagina. The results revealed that treatment with mesenchymal stem cells facilitated and promoted the regeneration of the vaginal wall (Janssen et al., 2019). However, due to the challenges associated with using stem cells—such as their limited availability, invasive harvesting methods, and the risk of tumor formation—new solutions are suggested (Balistreri et al., 2020).

Another innovative approach is the use of blood derived materials in conjunction with tissue-engineered constructs. Recent research has highlighted the benefits of using growth factor-loaded scaffolds to accelerate the healing of pelvic tissues and improve functional outcomes (Goonoo and Bhaw-Luximon, 2019; W et al., 2022; Jia et al., 2018).

One exciting development in this area is the use of autologous platelet-rich plasma (PRP), which enhances tissue repair and regeneration. Autologous PRP is derived from the patient’s blood and contains a high concentration of platelets, which release critical growth factors and cytokines for tissue healing. Recent studies have shown promising results in using PRP to treat various PFDs, including stress urinary incontinence and pelvic organ prolapse by stimulating cell proliferation, angiogenesis, and the synthesis of extracellular matrix components, promoting the regeneration of weakened or damaged tissues (Braga et al., 2024; Kurniawati et al., 2023; Prodromidou et al., 2022a; Prodromidou et al., 2022b). Recently, Kurniawati et al. published a systematic review analysis of 600 women who suffered from sexual dysfunction, perineal trauma, vulvovaginal atrophy, stress urinary incontinence, vesicovaginal, fistula, perineal rupture, and pelvic organ prolapse, and they received PRP therapy for this purpose (Kurniawati et al., 2023). They showed PRP had positive impacts on these disorders and suggested that PRP can be used to manage pelvic floor disorders (Kurniawati et al., 2023).

Similarly, Dankova et al. in another systematic review showed that PRP injections had been found to improve the structural integrity and function of the pelvic floor muscles and connective tissues, leading to better clinical outcomes in the treatment of female sexual dysfunction and stress urinary incontinence (Dankova et al., 2023).

Recent research has demonstrated the effectiveness of PRP-infused scaffolds in promoting the repair of pelvic floor defects in animal models, paving the way for similar applications in humans (Liu et al., 2023; Liu et al., 2022; Ávila et al., 2016; Medel et al., 2015). Incorporating PRP into tissue-engineered constructs or combined with stem cells or differentiated cells is another promising approach that further enhances its therapeutic potential (Medel et al., 2015; Paganelli et al., 2023). Paganelli et al., in a comprehensive review study of the combination therapy of PRP and adipose tissue stem cells therapy for genital lichen sclerosus, demonstrated significant improvements in lichen sclerosus-related symptoms, tissue tropism, and histological features (Paganelli et al., 2023). However, despite these advantages, the variability in PRP formulations and preparation methods resulted in different therapeutic effects on PFDs. Therefore, several challenges remain in the application of tissue engineering scaffolds in standardizing treatment protocols and ensuring reproducible results (Kurniawati et al., 2023).

Recently, our team has focused on establishing an autologous novel human blood-derived scaffold (hBDS) for the improvement of pelvic floor disorders, and in the current study, we studied and compared the *in vivo* biocompatibility and biodegradation by implantation in the subcutaneous, abdominal wall, and back muscle of mice. These murine models were chosen to evaluate the scaffold’s interaction with living tissue, offering insights into its potential for clinical applications in humans.

## Materials and methods

Sigma Aldrich (London, UK) provided all materials and reagents, otherwise mentioned in the text.

### Scaffold preparation

The human blood-derived scaffold was prepared according to the protocol of Smartfem Medical Technology Pty Ltd. patent (International Publication Number: WO 2023/028651 Al) (Behnia-Willison, 2022). Briefly, following informed consent obtained under the guidelines of the Ethics Committee of the Medical Faculty of Tarbiat Modares University (ethics reference code: IR.MODARES.AEC.1402.012), blood samples were collected individually, from healthy men (*n* = 5) and women (*n* = 2), aged 26–63 years, into two PRP test tubes (Sure Cell, Melbourne, Australia). The resulting scaffold was washed with phosphate-buffered saline, dissected into several fragments, and used for transplantation in three experiments.

### Animal

Male adult Naval Medical Research Institute (NMRI) mice, weighing 25–30 g on average (*n* = 66), were housed in the university animal facility under controlled conditions (12-h light/dark cycle at 22 °C ± 2 °C and 40%–50% humidity). The mice were randomly divided into three study groups: Experiment I, Experiment II, and Experiment III. All experimental protocols were approved by the ethics committee of Tarbiat Modares University (ethical reference number: IR.MODARES.AEC.1402.012).

### Study design

In this experimental study, xenotransplantation of the human blood-derived scaffold (hBDS) was performed subcutaneously, on the back muscle and abdominal wall in experiments I-III, respectively. Experiment I focused on assessing the *in vivo* biodegradation of the scaffold and the host response morphologically over 6 weeks. Hematoxylin and eosin (H&E) staining was used for histological examination, and Masson’s trichrome staining was applied to investigate collagen fiber changes. Immunohistochemistry for the CD136 marker was conducted to evaluate vascularization. Experiments II and III aimed to compare the effects of suturing the scaffold at multiple points on the abdominal wall and back muscle to prolong its biodegradation time and assess scar formation around the transplantation site.

### Experiment I: xenotransplantation of hBDS on the mouse subcutaneous

In this experiment, hBDSs were prepared from donor samples (*n* = 6) and cut into 3 × 3 mm^2^ pieces for surgical procedures (Supplementary Figures S1A, B).

### Surgical procedure

Adult male mice (*n* = 33) were anesthetized with intraperitoneal injections of ketamine (80 mg/kg) and xylazine (5 mg/kg). The dorsal skin of each mouse was shaved, and a small incision (5 mm) was made on the back. The hBDS fragments were inserted subcutaneously into the incision, followed by closure and suturing of the skin (Supplementary Figures S1C–E).

A sham operative group (*n* = 6) underwent the same anesthesia and skin manipulation procedure but without scaffold transplantation.

### Post-operative care

After surgery, the mice recovered under controlled conditions in the animal facility and were monitored throughout the study period. They were sacrificed by cervical dislocation at intervals of 72 h and weekly up to 6 weeks post-transplantation. Skin samples were harvested from the transplantation sites for morphological and immunohistochemical analyses.

### Light microscopy study of the recovered tissues

Samples collected from the subcutaneous transplantation site and the sham operative group (*n* = 39 total) were fixed in 10% formalin and dehydrated through a series of ascending ethanol concentrations (70%, 90%, and 100%). Following dehydration, the samples were cleared in xylene and embedded in paraffin wax. Sections were then cut at a thickness of 5 μm.

For histological staining, the tissue sections were deparaffinized, hydrated, and stained with hematoxylin and eosin (H&E) for routine morphological analysis. Additionally, another series of tissue sections were stained using Masson’s trichrome method to analyze collagen fiber and stromal changes. Finally, the stained tissue sections were examined under light microscopy.

### Evaluation of morphological changes in recovered tissue sections

For analysis under light microscopy, at least 6 tissue sections from each sample (*n* = 33 mice) at different intervals were randomly collected, and 4 microscopic fields at original magnification of 400 (X 400) were evaluated as follows: The presence and resorption of hBDS were scored as “+” and “–”. The intensity of scaffold biodegradation within different tissue sections was scored from “+”to “++++” indicating very low, low, moderate, and high degradation. The intensity of host fibroblasts around the transplanted scaffold and their penetration within the scaffold thickness were scored from “+” to “++++”, representing low (>25%), moderate (25%–75%), and high (<75%) levels. The infiltration of inflammatory cells such as white blood cells, lymphocyte, and macrophages around the transplant site was graded from 0 to ++++ indicating weak, moderate, and high infiltration, respectively. The formation of collagen fibers and blood vessels around hBDS was scored as negative (−) or positive (+).

### Statistical analysis

These qualitative data were calculated across multiple samples at each sampling time, and their averages were summarized. The non-parametric statistical analysis using the Kruskal–Wallis test and Dunn’s multiple was used to compare morphological changes during different time courses after transplantation.

### Immunohistochemistry for CD31

The tissue sections were collected from recovered samples one and 2 weeks after transplantation (*n* = 3 per week) and mounted on coated glass slides. The sections were deparaffinized in xylene for 20 min, followed by hydration in descending concentrations of ethanol and distilled water. Endogenous peroxidase activity was quenched by incubating the slides in a3% H_2_O_2_ solution for 10 min at room temperature. Subsequently, the samples were blocked with a blocking buffer for 1 h, then incubated with an anti-mouse CD31 primary antibody (Abcam; 28364, USA; diluted 1:200) for 1.5 h. After thorough washing, the sections were incubated with a peroxidase-conjugated secondary antibody (Santa Cruz Biotechnology, USA; diluted 1:200) for 30 min. Finally, the slides were developed with diaminobenzidine and examined under a light microscope.

### Experiment II: xenotransplantation and suturing of hBDS on the mouse abdominal wall

The hBDS (*n* = 3) were cut into fragments measuring 7 × 7 mm^2^ (Supplementary Figure S1E). Adult male mice (*n* = 12) were anesthetized with ketamine and xylazine, as previously described in Experiment I. The lateral surface of the animal skin was shaved, and a 1-cm incision was made through both the skin and abdominal wall thickness. One hBDS fragment was placed inside the abdominal wall (parietal peritoneum) and sutured at this site using a strand of PDS 6/0 at 10–12 points. Subsequently, all layers were closed and sutured (Supplementary Figures S1F–I).

After recovery, the mice were housed under controlled conditions and monitored for 4 weeks. Weekly the mice were sacrificed (*n* = 3) by cervical dislocation, and samples were collected from the transplantation site on the abdominal wall. These samples were fixed and processed for morphological staining, including H&E and Masson’s trichrome staining, as described in the previous sections. The statistical analysis of morphological changes in recovered tissue sections was carried out in a similar manner to Experiment I, as described earlier.

### Experiment III: xenotransplantation and suturing of hBDS on the mouse back muscle

Another series of hBDS (*n* = 3) was prepared, washed in PBS, and dissected into fragments measuring 7 × 7 mm^2^ (Supplementary Figure S1J). Male mice (*n* = 12) were anesthetized with ketamine and xylazine, following the procedure described in Experiment I. The lower lumbar area of the animal’s skin was shaved, and a 1-cm incision was made to expose the back muscle. Each scaffold fragment was transplanted into the back muscle of the mice and sutured with 10–12 points, similar to Experiment II. Subsequently, the skin was closed and sutured (Supplementary Figures S1K–N). For morphological studies, tissue samples were collected weekly for 4 weeks, then histomorphological changes of recovered tissue sections were analyzed by non-parametric tests such as the other studied groups.

## Results: morphological observations after transplantation of scaffold subcutaneously

**FIGURE 1:**
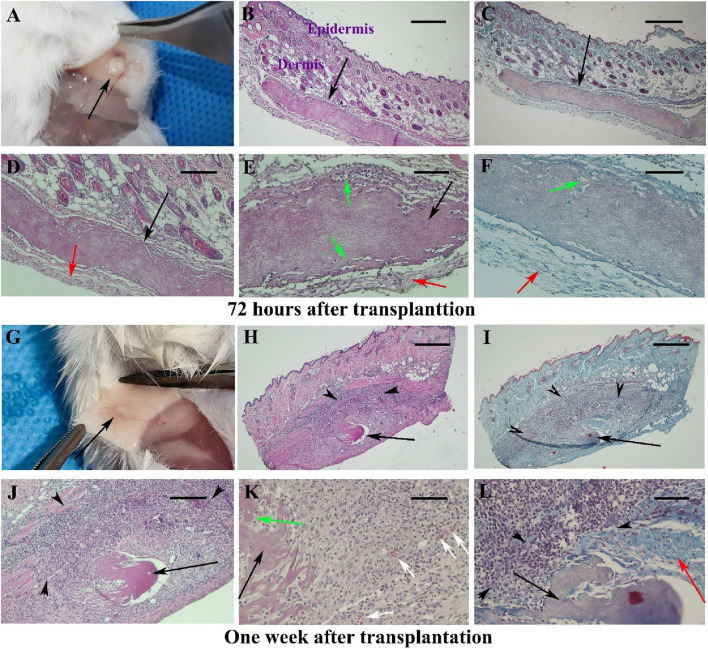
Observations of skin samples 72 h **(A–F)** and 1 week **(G–L)** after surgery in Experiment I at different magnifications. The gross appearance of mouse skin is shown in **(A,G)**, with the scaffold appearing white and indicated by black arrows. Representative tissue sections stained with hematoxylin-eosin (first and second columns) and Masson’s trichrome (third column) are illustrated. The scaffold is identified as acidophilic (black arrows), with infiltration of fibroblasts around it marked by black arrowheads. Red arrows indicate boundary collagen fibers around the scaffold and the degradation of the scaffold at the periphery, along with the penetration of host fibroblasts. Green arrows show the penetration of host fibroblasts at the scaffold’s periphery, while white arrows denote newly formed blood vessels. Scale bars: **(B, C, H, I)**; 400 μm, **(D, J)**; 200 μm and **(E, F, K, L)**; 100 μm.

All animals survived after surgery and recovery time. The gross 72-h post-transplantation observations revealed no inflammatory reaction and no sign of infection at the implantation site. The scaffold is visible and appears white under the mouse skin (Figure 1A; black arrow). The histopathological changes around the scaffold transplantation site over 6 weeks after surgery are summarized in Supplementary Table S1. Representative micrographs from samples collected at 72-h post-transplantation is shown in Figures 1B–F. By H&E and Masson’s trichrome staining, hBDS was observed as an eosinophilic material under light microscopy. The scaffold border appeared intact, surrounded by a thin band of connective tissue composed of delicate collagen fibers and fibroblasts (green arrow). Some spaces around the fibroblasts within the scaffold are indicative of scaffold degradation (Figures 1C,F; green arrow). Moderate inflammatory reaction (43.75%) and fibroblast penetration within the scaffold (43.75%) were noted. Scaffold biodegradation was weak (37.5%), with no evidence of newly formed blood vessels or scar formation. One-week post- transplantation, the scaffold was visible as white in subcutaneous samples (Figure 1G; black arrow). Microscopic evaluation (Figures 1H–L) revealed hBDS presence in 71.5% of samples (5 of 7 cases), with irregular scaffold margins. Biodegradation was high (75%), accompanied by a significant increase in fibroblast population around the scaffold (black arrowheads), demonstrating active invasion (85.7%) and degradation. Inflammatory reaction decreased to 25%, and neovascularization was observed at scaffold boundaries in 71.5% of samples (Figure 1K; white arrow). Masson’s trichrome staining showed dense collagen deposition (red arrow) indicative of scar formation in 85.7% of samples.

**FIGURE 2:**
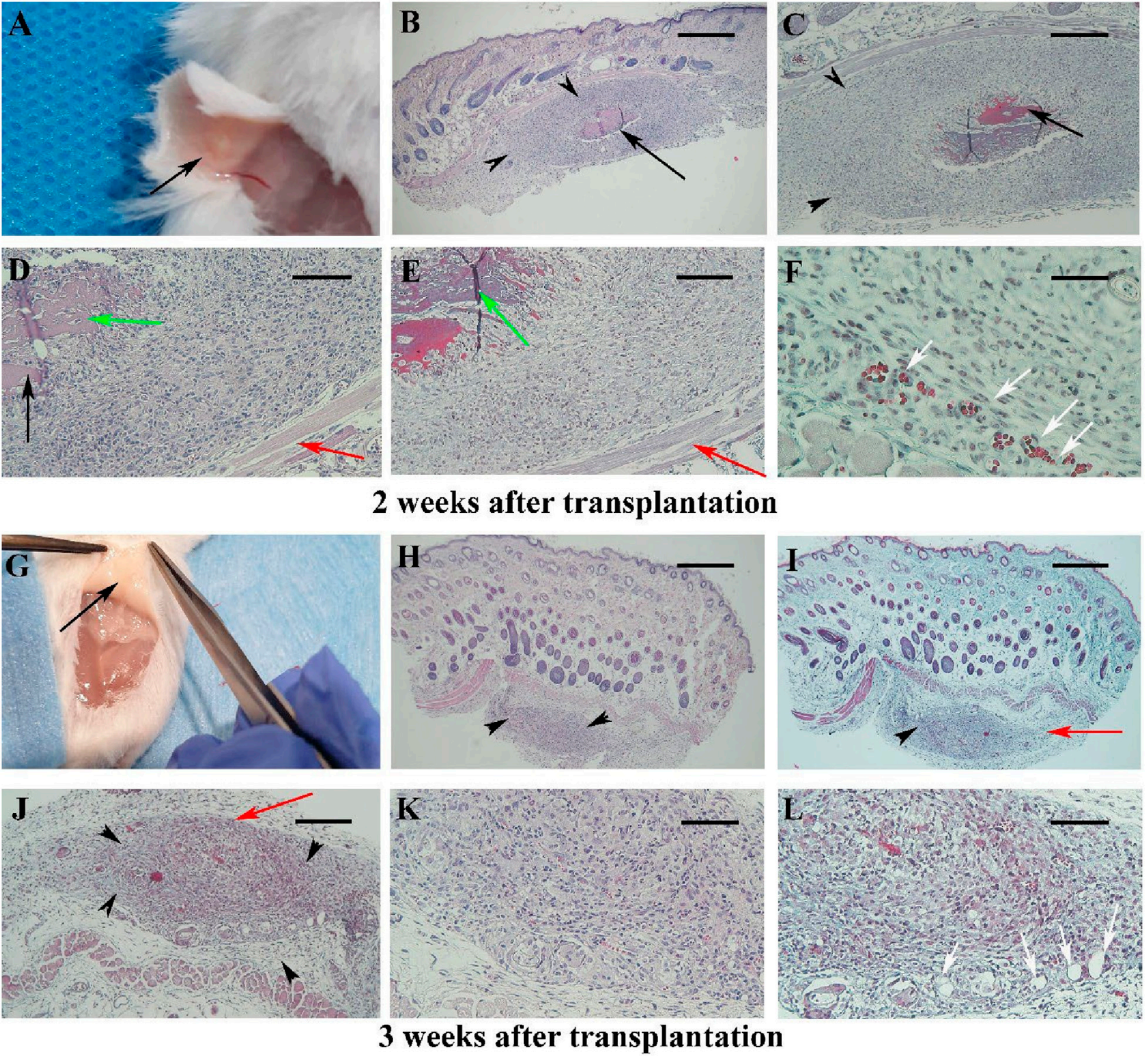
The morphology of the mouse skin 2 and 3 weeks after surgery in Experiment I at different magnifications is presented in **(A–F)** and **(G–L)**, respectively. Micrographs stained with hematoxylin and eosin are shown in **(B, D, H, J, K)**, while those stained with Masson’s trichrome are shown in **(C, E, F, I, L)**. The scaffold is barely visible after 2 weeks [**(A)**; black arrow] and is not detected macroscopically at week 3 **(G)**. Under light microscopy, black arrows indicate the scaffold, black arrowheads show the infiltration of fibroblasts around the scaffold, and red arrows point to collagen bundles around the scaffold. Green arrows indicate the penetration of fibroblasts within the scaffold. Newly formed blood vessels are demonstrated by white arrows. Scale bars: **(B, H, I)**; 400 μm, **(C, J)**; 200 μm and **(D, E, K, L)**; 100 μm.

By week two post-transplantation, the scaffold was barely discernible macroscopy (Figure 2A) in nine analyzed samples. The hBDS size was reduced (33.33%) and its degradation was extensive (88.88%) (black arrow). Intense fibroblast infiltration was noted around the scaffold (69.4%; black arrowheads) with fibroblast penetration into the scaffold (green arrow). Some neovascularization was observed (33.33%; white arrow), while the inflammatory reaction was weak (25.27%). Masson’s trichrome staining indicated collagen bundle presence (red arrow) and scar formation in 66.66% of samples (Figures 2E,F).

By week 3 post-transplantation, hBDS was completely resorbed and absent in almost all samples (Figure 2G), confirmed microscopically with 100% disappearance. Fibroblast infiltration persisted (25%), with minimal neovascularization (18.3%), collagen deposition (red arrow), and scar formation (16.3%) observed (Figures 2H–L).

**FIGURE 3:**
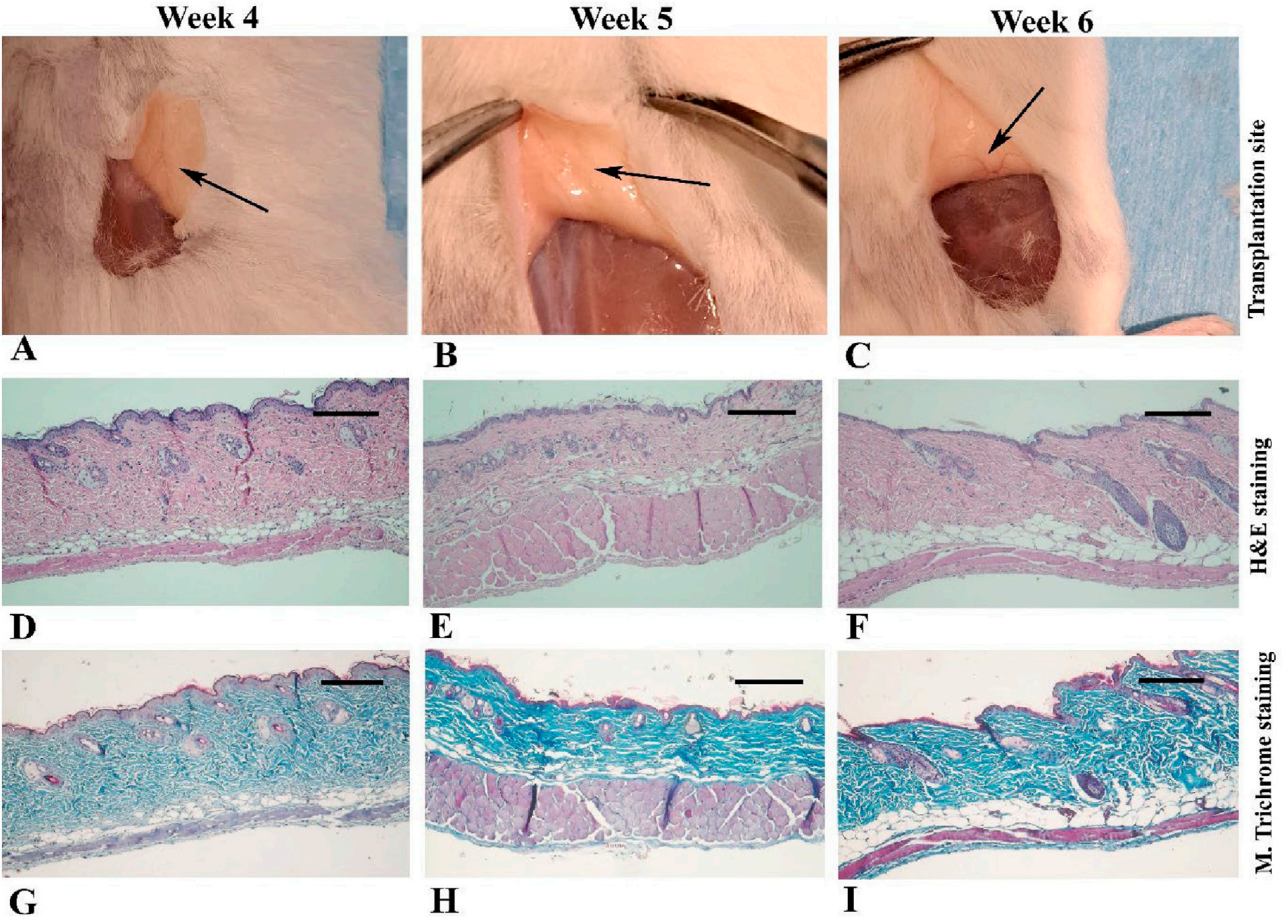
Gross appearance and light microscopic observations of mouse skin 4–6 weeks after transplantation in Experiment I. In the first row **(A–C)**, black arrows indicate the transplantation site where the scaffold is no longer visible. The second row **(D–F)** presents micrographs stained with hematoxylin and eosin, while the third row **(G–I)** shows the corresponding sections stained with Masson’s trichrome. Scale bars: D–I, 400 μm.

Between 4 and 6 weeks post-transplantation (Figures 3A–C), the normal dermal structure was observed microscopically (Figures 3D–I) and there were no signs of inflammation or fibroblast infiltration, it was similar to intact and sham operative control samples (Supplementary Figures S2).

### Statistical analysis of histopathological alterations during 6 weeks after subcutaneous transplantation of hBDS

**FIGURE 4:**
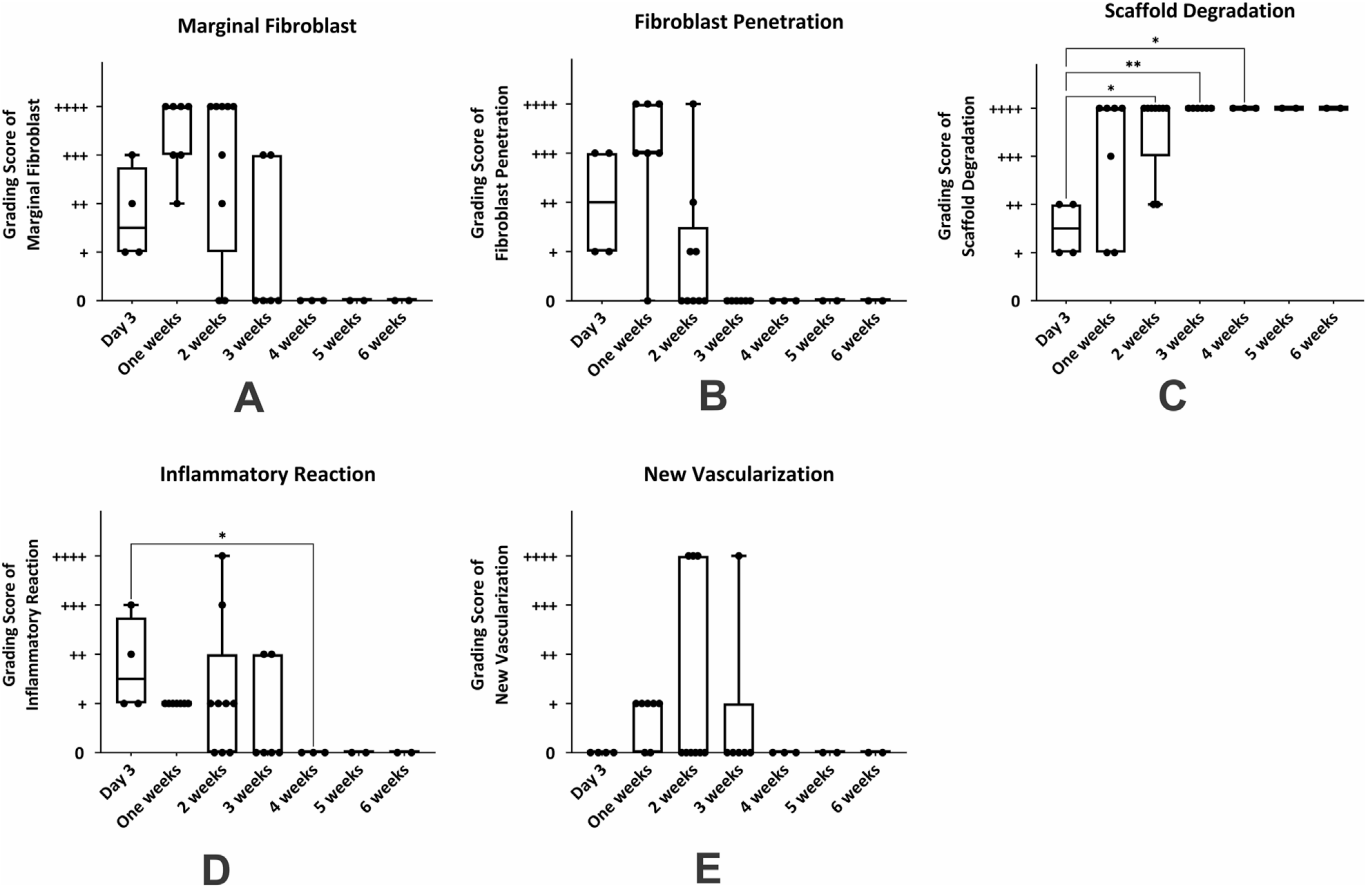
Non-parametric analysis of histological changes in mouse skin from day 3–6 weeks after subcutaneous transplantation of a human blood-derived scaffold. **(A)** marginal fibroblast infiltration; **(B)** fibroblast penetration; **(C)** scaffold degradation; **(D)**: inflammatory cells; **(E)** new vascularization. Significant differences with day 3 (*P < 0.05; **P < 0.01).

The summary of histological changes across all samples is depicted in Figures 4A–E. Analysis of the grading scores of marginal fibroblasts at different time points revealed that the highest presence of these cells occurred during the first and second weeks. From the third week onward, a decreasing trend was observed, from the fourth to sixth weeks. This pattern indicates an initial intense cellular response during the early stages, followed by a gradual decline over time Figure 4A. The maximal fibroblast infiltration and penetration were observed 1 week after surgery, declining thereafter by week two post-transplantation. Afterward, the infiltration gradually decreased, reaching very minimal levels from the third week onward and remaining almost constant. The non-parametric statistical analysis using the Kruskal–Wallis test and Dunn’s multiple comparisons results indicated that the differences in the mean marginal fibroblasts, and fibroblast infiltration and penetration between day three and the other time period were not statistically significant (Figure 4B; *p* > 0.05).

The boxplot results demonstrated a significant increase in scaffold degradation scores over time. On day three, the level of scaffold degradation was minimal, while from the second week onward, its degradation nincreased. By the fourth to sixth weeks, biodegradation of scaffold was complete. Dunn’s test results indicated statistically significant differences between day three and the second week (*p* = 0.0159), third week (*p* = 0.003), and fourth week (*p* = 0.0196). These findings confirm that scaffold degradation began gradually and significantly from the second week, reaching its peak by the fourth week. Conversely, the differences between day three and the first, fifth, and sixth weeks were not statistically significant (Figure 4C; *p* > 0.05).

The results showed that the inflammatory response was high on day three and significantly decreased in the first week, although this reduction was not statistically significant. In the second and third weeks, despite a relative increase in the mean scores, no significant statistical difference was observed compared to day three. Notably, there was a significant reduction in the intensity of the inflammatory response during the fourth week, which was significantly lower than on day three (*p* = 0.0493). This decreasing trend continued after the fourth week in subsequent weeks. Findings indicated a transient and staged pattern of the inflammatory response, characterized by an initial peak followed by a gradual reduction and eventual subsidence (Figure 4D). The chart data indicated that the number of new vessels (neovascularization) increased from day three to the third week, reaching its peak in the second and third weeks then this trend declined, the degree of vascularization over this period did not show a statistically significant difference between these days (Figure 4E; *p* > 0.05).

### The immunohistochemistry observation

**FIGURE 5:**
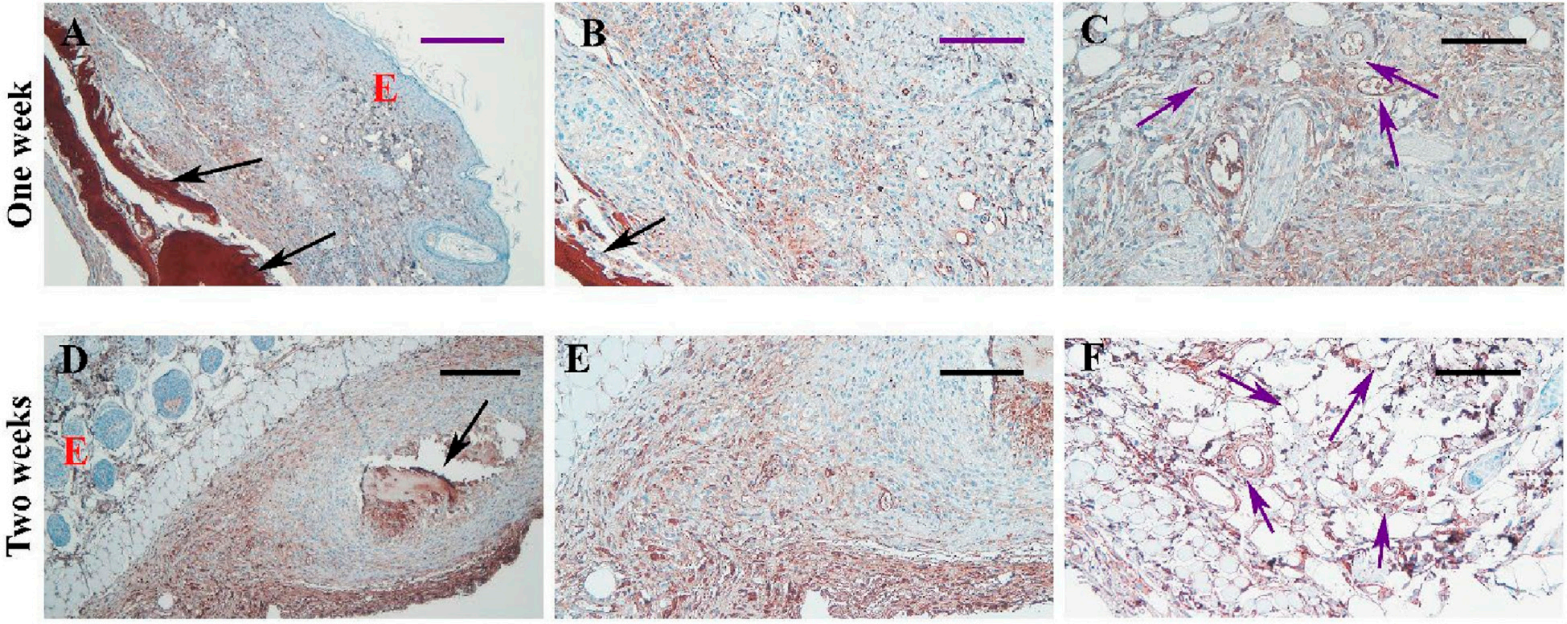
Immunohistochemistry for the CD31 marker in tissue sections of samples from Experiment I. Representative micrographs at different magnifications are shown for samples 1 week **(A–C)** and 2 weeks **(D–F)** after transplantation. Black arrows indicate the scaffold, and purple arrows point to blood vessels exhibiting a positive reaction to the CD31 marker (brown color). Scale bars: **(A, D)**; 200 μm, **(B, E)**; 100 μm and **(C, F)**; 50 μm.

We conducted immunohistochemistry using the CD31 antibody to specifically detect and confirm neovascularization, highlighting new small blood vessels as indicated by brown staining in Figures 5A–F, particularly prominent at one and 2 weeks after transplantation.

### Morphological observation of hBDS suturing on the peritoneum

**FIGURE 6:**
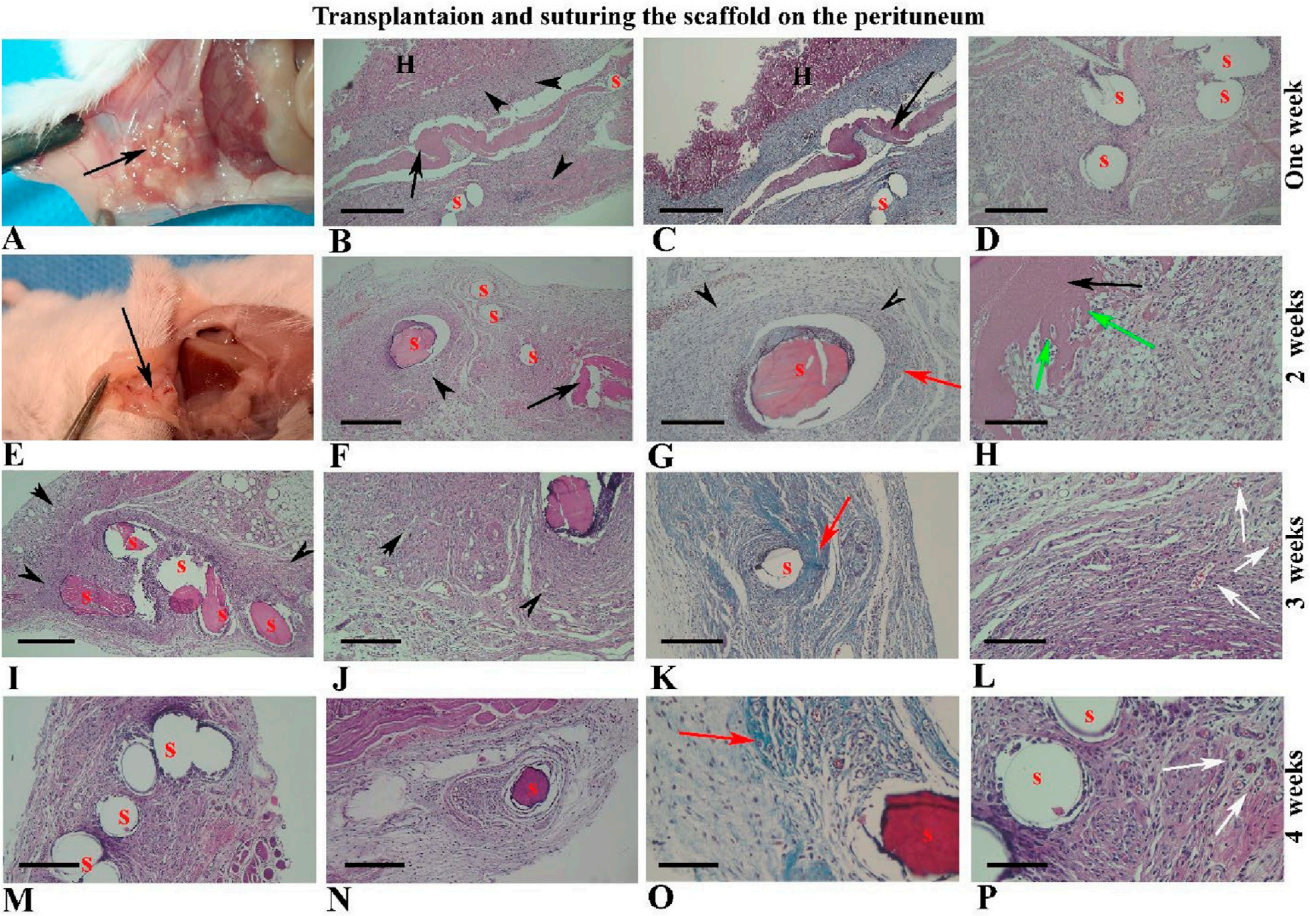
Morphological observations of human blood-derived scaffold suturing on the abdominal wall (peritoneum) from 1 to 4 weeks after surgery. The black arrows indicate the site of scaffold transplantation, visible in weeks one and two **(A–H)**, but not thereafter **(I–P)**. Sutures appear pinkish, with some detached and washed out from tissue sections, leaving hollow spaces (S). Intensive collagen fibers around the sutures and scaffold are highlighted in green by Masson’s trichrome staining (third column) and other images stained with hematoxylin and eosin. Black arrowheads and red arrows indicate fibroblast infiltration and collagen bundles around the scaffold and sutures, respectively. Green arrows show fibroblast penetration within the scaffold, while newly formed blood vessels are indicated by white arrows. Abbreviations: S: suture sites; H: hepatocytes. Scale bars: **(B, C)**, 400 μm, **(D, F, I, K, M, N)**; 200 μm, **(G, J, H)**; 100 μm and **(O, P)**; 50 μm.

The grading of histopathology changes of collected tissues during 4 weeks after suturing on the abdominal wall are summarized in Supplementary Table S2. After 1 week of suturing the hBDS on the interior part of the abdominal wall (lateral peritoneum), the scaffold was observed in all recovered samples (Figures 6A–D), while there were some adhesions in visceral organs such as the liver and intestine (Figures 6B,C). The scaffold appeared as a pinkish eosinophilic substance in tissue sections under the light microscope (Figures 6B–D; black arrow). Additionally, the sutures were pinkish, and in some instances, due to detachment and washout from tissue sections, the location of the sutures appeared as hollow spaces. Morphologically, the host reaction was similar to Experiment I, characterized by intensive infiltration of active fibroblasts and migratory cells around the transplanted scaffold and sutures.

Representative micrographs of tissue sections two weeks after suturing on the peritoneum are shown in Figures 6E–H. These images revealed that the scaffold size had reduced and it was observed in 66.66% of samples, while the sutures remained intact. The proportion of fibroblasts around the suture was high (83.33%) and some fibroblast cells were penetrated within the scaffold thickness (83.33%), creating spaces around themselves (Figure 6H; green arrow).

By 3 weeks after suturing on the peritoneum, the scaffold had completely disappeared and was not visible in tissue sections, while the infiltration of fibroblasts around the sutures remained prominent and high (91.66%; Figures 6I–L). Masson’s trichrome staining revealed distinctive collagen bundles around the sutures (red arrow), and it was more intensive on Week 3 (91.66%). By the fourth week after surgery, fibroblast infiltration around the sutures had declined (75%), as depicted in Figures 6M–P. New tissue vascularization was prominent around the sutures at two and 3 weeks after surgery (Figures 6L,P; white arrow).

### Statistical analysis of histopathological alterations during four weeks after suturing of hBDS on the peritoneum

**FIGURE 7:**
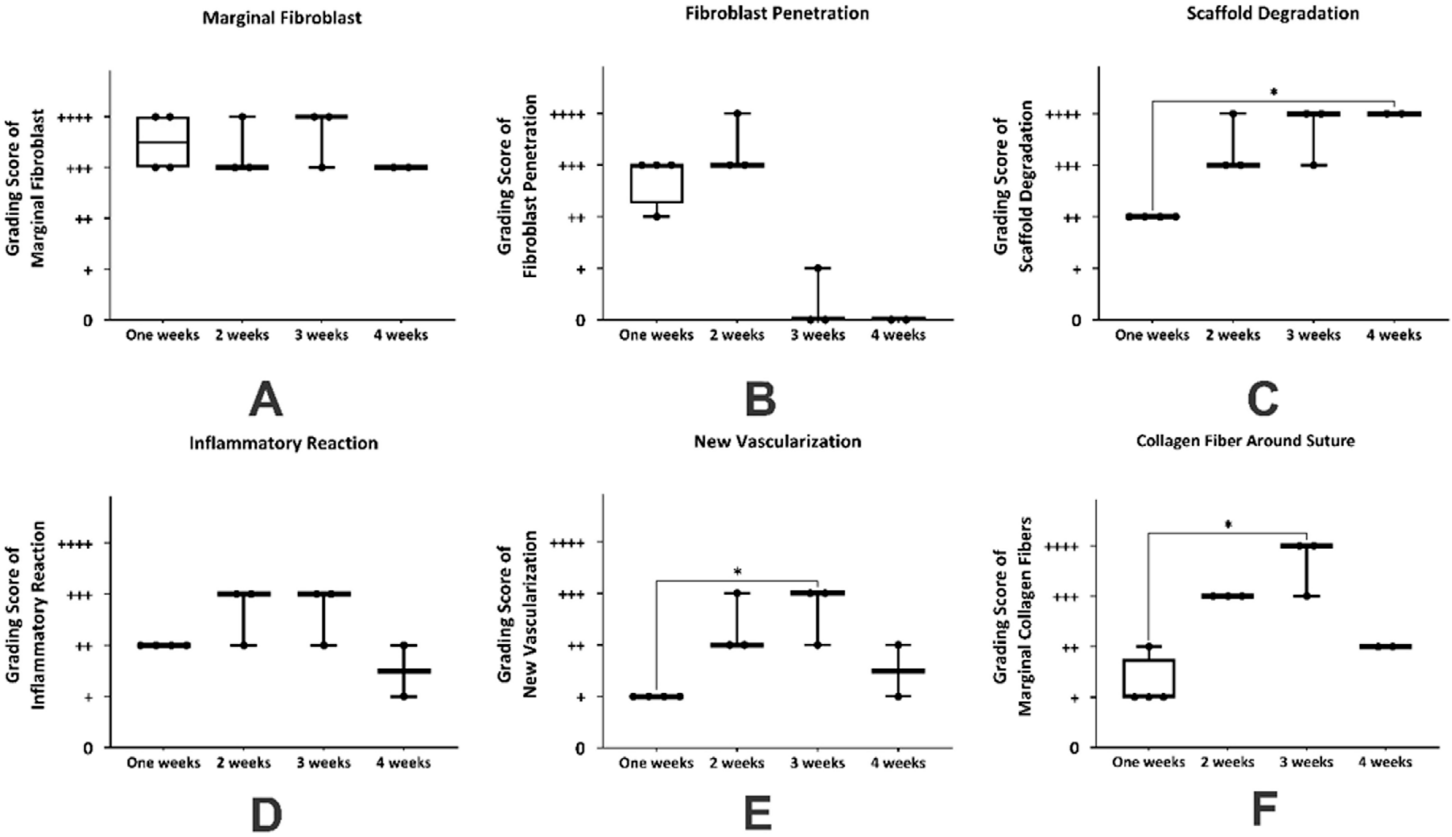
The statistical analysis of histological changes in mouse abdominal wall from week 1–4 after suturing the human blood-derived scaffold on the peritoneum. **(A)** marginal fibroblast infiltration; **(B)** fibroblast penetration; **(C)** scaffold degradation; **(D)** inflammatory reaction; **(E)** new vascularization, and **(F)** collagen fiber around suture. *Significant differences with day 3 (*p* < 0.05).

The summary of non-parametric analyses of morphological changes in tissue sections derived from suturing hBDS on the peritoneum is illustrated in Figures 7A–F. In the first week, marginal fibroblasts exhibited relatively high infiltration around the implantation site, while this amount decreased by week 4 (Figure 7A). Fibroblast penetration within the scaffold was active in weeks 1 and 2 after surgery, then declined and ceased by week 4. However, statistical analysis did not show any significant differences across the time courses (Figure 7B).

The biodegradation of the scaffold increased significantly from week 1 to week 3 and then stabilized at a high level. This trend aligns with the expected behavior of biodegradable materials, where the initial phase involves slower degradation and the middle-to-final phase involves acceleration toward maximum degradation. Statistical analysis showed no significant differences between week 1 and week 2, or between week 1 and week 3, whereas the difference between week 1 and week 4 was statistically significant (Figure 7C; *p* = 0.0315). The inflammatory reaction was high in week 1, then increased in weeks 2 and 3, and subsequently decreased in week 4. Statistical analysis did not reveal any significant differences among the weeks (Figure 7D). Graphical data indicate that new vessel formation increased from week 1, peaked at week 3, and declined in week 4. Non-parametric analysis showed a significant difference between weeks 1 and 3 (Figure 7E; *p* = 0.0246). Analysis of the intensity of collagen fiber around the suture showed that this variable initially improved and significantly increased from week 1 to week 3 (Figure 7F; *p* = 0.0107).

### Morphological observation of hBDS suturing on the muscle

**FIGURE 8:**
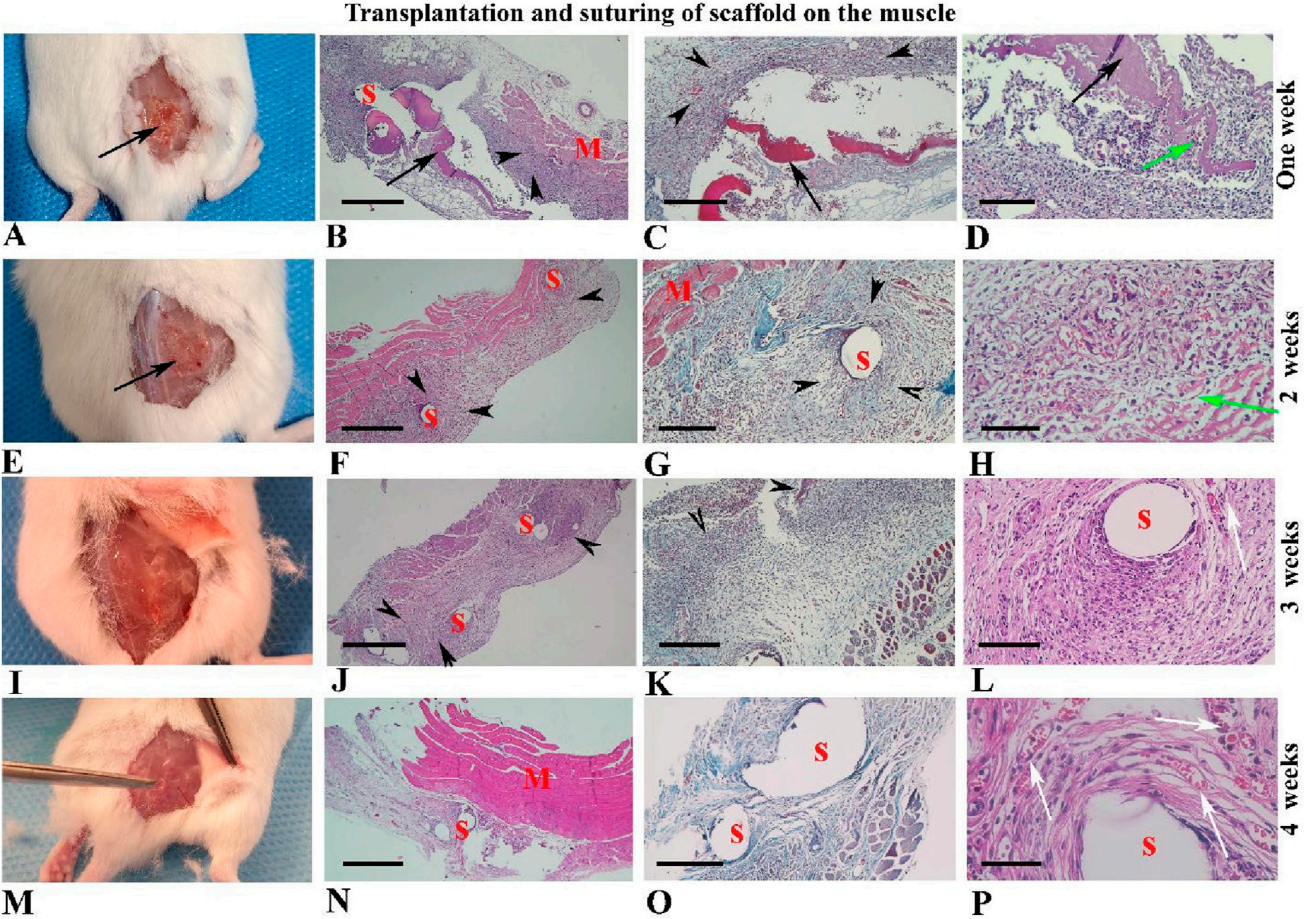
Morphological observations of human blood-derived scaffolds suturing on the back muscle **(A–P)**. Gross morphology of mouse samples is presented from 1 to 4 weeks after surgery in the first column **(A, E, I, M)**. Hematoxylin and eosin staining tissue sections with low and high-power magnifications are illustrated in the second and fourth columns, respectively. Micrographs related to Masson’s trichrome staining are shown in the third column. Black arrows indicate the scaffold transplantation site, visible in weeks one and two **(A–H)**, but not thereafter **(I–P)**. Red arrows highlight collagen fibers around the sutures and scaffold. Black arrowheads indicate fibroblast infiltration around the scaffold and sutures, while green arrows show fibroblast penetration within the scaffold **(D, H)**. Newly formed blood vessels are indicated by white arrows **(L, P)**. Abbreviations: S: suture sites appear hollow spaces; M: muscle tissue. Scale bars: **(B, F, J, N)**, 400 μm, **(C, G, K, O)**; 200 μm, **(D, L)**; 100 μm and **(H, P)**; 50 μm.

For this part of the study, 12 samples were assessed, and the representative micrographs of the samples over 4 weeks after suturing are depicted in Figures 8A–P. As shown, the scaffold was visible for up to 2 weeks (Figures 8A,E; black arrow), but it disappeared thereafter by week 3 (Figures 8I,M). The grading of these histological alterations is shown in Supplementary Table S3.

### Statistical analysis of histopathological alterations during four weeks after suturing of hBDS on the muscle

**FIGURE 9:**
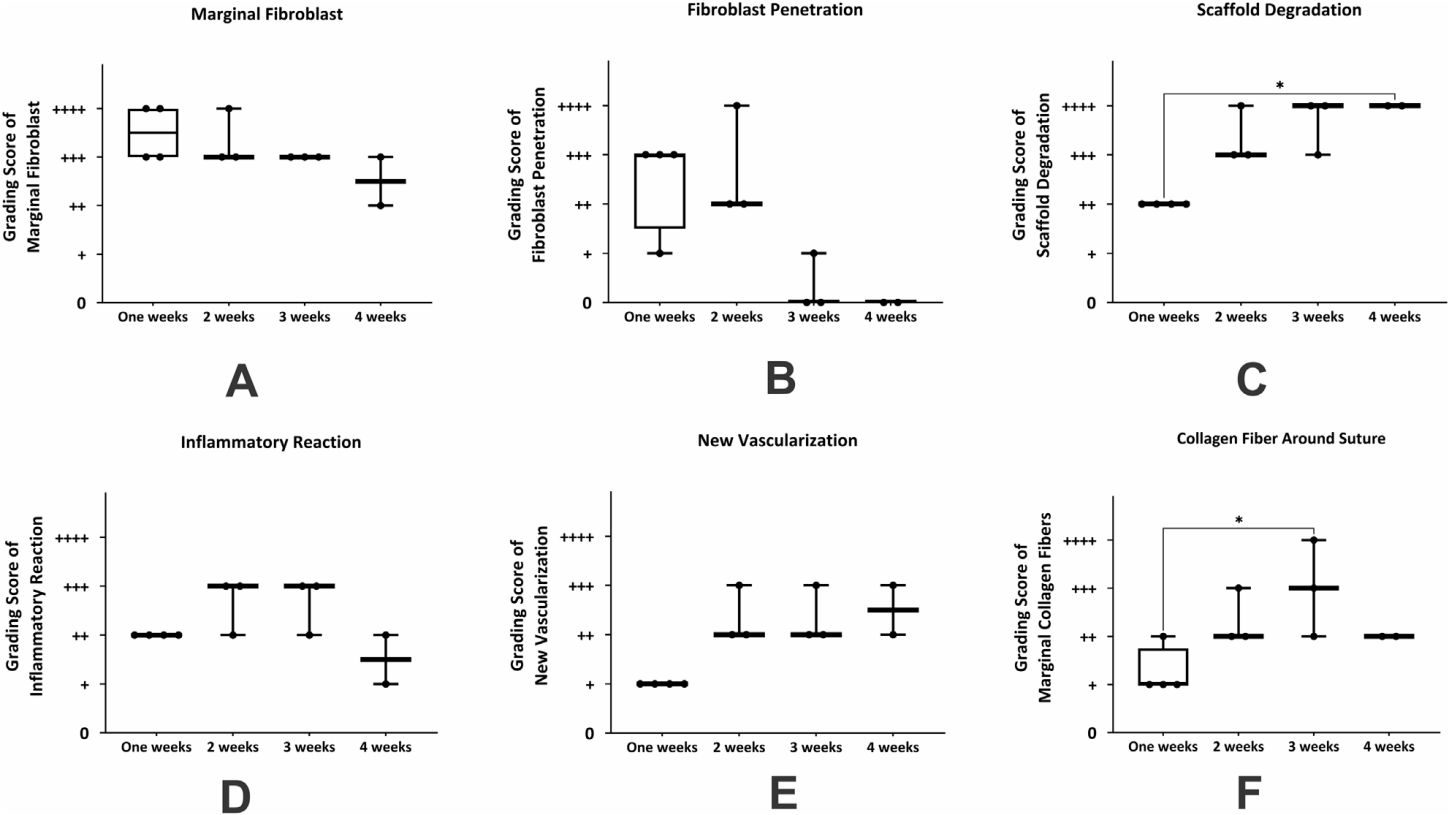
The summary of non-parametric analysis of histological alterations around the site of suturing of the human blood-derived scaffold on the back muscle during 4 weeks. **(A)** marginal fibroblast infiltration; **(B)** fibroblast penetration; **(C)** scaffold degradation; **(D)** inflammatory reaction; **(E)** new vascularization, and **(F)** collagen fiber around suture. *Significant differences with day 3 (p < 0.05).

The assessment of marginal fibroblasts across different time intervals showed that the highest proportion of marginal fibroblasts and the greatest fibroblast penetration within scaffolds occurred in week 1 after transplantation and then decreased gradually through week 4. This pattern aligns with the classic phases of tissue repair, where cellular infiltration is higher in the initial stages and is followed by tissue remodeling and organization. However, these differences were not statistically significant (Figures 9A,B). Results indicate that scaffold degradation, the intensity of the inflammatory response, and the formation of new vessels increased markedly from week 1 to week 3. Nevertheless, there were no significant differences among these parameters across the time courses (Figures 9C–E).

Analysis of collagen fiber intensity around the sutures showed that this variable rose from week 1 to week 3, then decreased in week 4. A statistically significant difference was observed between week 1 and week 3 (Figure 9F; *p* = 0.0313).

## Discussion

The primary purpose of using scaffolds in regenerative medicine is to provide a well-organized matrix for cell adhesion, proliferation, and differentiation toward the formation of new tissue or the repair of damaged tissue. Therefore, the scaffold degradation rate should match the rate of new tissue formation. In this study, we investigated the *in vivo* biocompatibility and degradation of a hBDS in a mouse model. Consistent with our *in vitro* data, our observations confirmed the biodegradation capacity of this scaffold. The maximum degradation of the scaffold was observed 2 weeks after implantation and had completely resorbed on week 3. This observation is consistent with previous work on the plasma-rich fibrin biodegradation time that has some similarities with our presented scaffold (Miron et al., 2000; Gheno et al., 2021; Fujioka-Kobayashi et al., 2021). These *in vivo* investigations also showed the stability of blood-derived biomaterials in a short time of 2 weeks (Miron et al., 2000; Gheno et al., 2021; Fujioka-Kobayashi et al., 2021).

The light microscopic observation showed a large population of fibroblast infiltration around the scaffold implantation site. The fibroblasts are bifunctional cells during tissue repair, mainly in extracellular matrix formation. Masson’s trichrome staining confirmed new fibrillar collagen biogenesis around the scaffold. In addition, fibroblasts can secrete proteolytic enzymes to digest protein and glycosaminoglycan molecules of the extracellular matrix (Kurniawati et al., 2023; Chu and Quan, 2024; Aguado-Alvaro et al., 2024). The spaces around the fibroblasts that penetrated within the scaffold indicate the enzymatic activity of these cells. The infiltration and proliferation of fibroblasts around the scaffold could be a response to the action of inflammatory cells, such as macrophages, which release chemotactic cytokines (Velnar et al., 2009).

Our ultrastructural study by scanning electron microscopy showed that many platelets are trapped in the inner parts of the applied scaffold. These platelets contain several types of growth factors such as platelet-derived growth factors (PDFG), transforming growth factor beta, epithelial growth factor, and vascular endothelial growth factor (VEGF) that have positive effects on the proliferation and migration of fibroblasts to the site of the scaffold (Dos Santos et al., 2021). It appears that these observations may relate to the effects of broad growth factors present within scaffolds, originating from blood-derived sources—particularly from blood cells such as platelets or from blood plasma. Previous research in this area has similarly shown that PRP contains growth factors such as PDGF and VEGF. Several key human platelet-derived growth factors, including PDGF and VEGF, retain their biological activity in murine systems (Dos Santos et al., 2021; Gerber et al., 1999), which likely contributed to the fibroblast infiltration and angiogenesis observed in the current study.

PDGF can play a role in cellular proliferation and in accelerating tissue repair. Similarly, VEGF promotes angiogenesis, and our observations indicate a significant increase in neovascularization during the second and third weeks. The formation of these new blood vessels, together with an increased number of resident fibroblasts at the site of transplantation, could make a meaningful contribution to advancing tissue repair. However, after the scaffold degrades, the number of fibroblasts decreased like tissue healing, and the tissue at the site of scaffold implantation retains its normal morphology 4 weeks after surgery. Although we did not directly quantify the concentration of growth factors within the fabricated scaffold, its composition is biologically comparable to PRP, as both are blood-derived products enriched in platelets. Given that platelets are a well-established source of numerous growth factors, it is reasonable to infer that the scaffold also contains a similarly high concentration of these bioactive molecules (Dos Santos et al., 2021). Moreover, in many investigations, the application of PRP evidenced significant improvement in the outcome of pelvic organ prolapse treatment (Dankova et al., 2023; Prodromidou et al.; Kurniawati et al., 2023; Saraluck et al., 2024), and an increase in the collagen content of recovered tissue was observed (Abuaf et al., 2016). In addition, in animal models, similar results showed PRP regulates tissue reconstruction (Dias et al., 2021; Boru et al., 2022).

The inflammatory reaction around the scaffold was seen morphologically a few days after implantation, and the presence of foreign body giant cells was very rare, and it was seen just in one sample 2 weeks after surgery. This result confirmed the biocompatibility of the applied scaffold. It is suggested that the applied scaffold contains only a minimal number of nucleated donor cells, and this low level of WBCs is insufficient to trigger an active immune response. Furthermore, the immunomodulatory properties of PRP, as demonstrated in other studies, may also contribute to this effect. It is concluded that during the degradation of the scaffold in three groups of study there was no harmful byproduct that caused the chronic inflammatory reaction. The acute inflammatory response is necessary for the recruitment of fibroblasts to reconstruct the damaged tissue (Martin and Leibovich, 2005) and we observed a low inflammatory reaction around the implantation site 2 weeks after surgery and it declined after that due to the nature of the scaffold. It is a natural substance derived from blood components and is very similar to PRP, and similarly, the anti-inflammatory effect of PRP has been demonstrated before (Belebecha et al., 2020). This is an advantage of the applied scaffold in the present study. Increased inflammation can lead to excessive fibroblast activity, resulting in severe scar tissue formation which changes the stiffness and density of the connective tissue in the implantation site (Martin and Leibovich, 2005; Tedesco et al., 2020). Clinically, this stiffness is undesirable and can cause problems for the patient, similar to what has been observed with other synthetic or natural scaffolds (Tedesco et al., 2020).

Moreover, we observed morphologically and by immunohistochemistry small and newly formed blood vessels around the scaffold implantation side, are signs of neovascularization. This is more prominent around 2 weeks after transplantation due to the release of some angiogenic growth factors by blood-derived scaffold or some inflammatory cells. The vascular endothelial growth factor has a critical role in angiogenesis and is one of the growth factors that release from the platelet granules (Naderi et al., 2020).

On the other hand, our study findings showed that the *in vivo* biodegradation time of the scaffold was short, with maximum degradation occurring within 2 weeks after implantation and this degradation time was comparable to other natural scaffolds (Boennelycke et al., 2011; Ramanah et al., 2010; Armitage et al., 2012), the improvement of biodegradation time is necessary and motivates further study. For pelvic floor disorder treatment, the scaffold would need modifications to enhance its strength and prolong degradation time, given the dense connective tissue structure of the pelvic floor.

Additionally, we used absorbable suture threads to attach the scaffold to the peritoneal wall and back muscle, similar to clinical practice. Results showed higher fibroblast infiltration and collagen formation around the suture compared to the non-suture group, with no differences between the location of the suture (abdominal wall or back muscle). The suture thread acted as an additional scaffold, with a longer degradation time leading to greater fibroblast density and function. This is in line with our clinical observations, in 85% of 400 female patients with pelvic floor disorders showed reduced symptoms over 2 years after suturing this autologous scaffold on the vaginal wall (unpublished data).

These promising results contrast with studies using synthetic scaffolds, which often show chronic inflammation and increased type I collagen density, leading to excessive tissue hardness and scar tissue formation (O’Shaughnessy et al., 2022). For instance, in an experimental study, a fully absorbable scaffold (poly-4- hydroxybutyrate) and permanent mesh (polypropylene) were implanted to rabbit abdomen and vagina. Their results demonstrated chronic inflammation and an increase in type I/III collagen in all specimens at 3- and 9-months post-implantation (O’Shaughnessy et al., 2022). We hypothesize that the delayed absorption of suture threads could be a suitable alternative to synthetic mesh. However, for surgical application in PFDs, scaffolds need prolonged maintenance and stability *in vivo*, and it can potentially be improved by further investigations.

In addition to the findings presented in this study, it is important to place the performance of the human blood-derived scaffold (hBDS) in the context of current surgical materials used for pelvic floor repair. Permanent polypropylene meshes, although mechanically robust, are associated with long-term foreign body reactions, chronic inflammation, and mesh-related complications such as erosion and pain (Maher et al., 2013; Cao et al., 2013). In contrast, fully absorbable polymer-based meshes degrade more rapidly and often demonstrate limited mechanical support over extended periods, which may compromise long-term repair integrity.

Our hBDS demonstrates a gradual degradation profile, minimal inflammatory response, and favorable tissue remodeling characteristics, potentially offering a middle ground between these two extremes. While the current study is limited to short-term *in vivo* evaluation, these features suggest that hBDS could address some of the complications seen with synthetic meshes while still providing adequate support during the critical healing period. Future studies directly comparing hBDS and synthetic meshes in clinically relevant large-animal or human models are warranted to confirm these advantages.

In another point of view, the created scaffold shows potential for other applications, such as a drug or cell delivery tool due to its blood-derived nature and short biodegradation time, more studies are needed to confirm this potential. In regenerative medicine with cell-based therapies, the blood- derived scaffold can serve as a temporary cell carrier to release and transplant a limited number of differentiated cells or undifferentiated stem cells into a defined location of the host tissue.

Open questions for future research concern additional translational aspects of the scaffold’s performance, including:

• Quantitative biomechanical assessments such as tensile strength and shear testing to evaluate scaffold integration and reinforcement in anatomically relevant pelvic sites.
• Extended implantation studies in larger animal cohorts to assess long-term tissue remodelling, scaffold–tissue interface behaviour, and durability of the repair.
• Quantitative biochemical assays (e.g., hydroxyproline content, cytokine profiling) performed in parallel with imaging and histological analyses, enabling multi-modal validation of scaffold performance.

These planned studies will provide a more comprehensive understanding of the scaffold’s mechanical and biological integration over extended periods and under clinically relevant loading conditions.

In conclusion, the novel human blood-derived scaffold demonstrated biodegradation and high biocompatibility. Its suturing on the abdominal wall or back muscle could effectively improve clinical symptoms, while further improvements are needed for its clinical application.

## Data Availability

The raw data supporting the conclusions of this article will be made available by the authors, without undue reservation.
