# Supplementary material for: In Vivo investigation of xenotransplanted human blood-derived scaffold into mice as a biodegradable construct for improvement of pelvic floor repair
Source: Front Bioeng Biotechnol. 2025 Sep 26;13:1627538. doi: 10.3389/fbioe.2025.1627538 (PMC12511874; doi:10.3389/fbioe.2025.1627538)
Supplement: Supplementary file 1 [file Supplementaryfile1.docx]

# Appendix A: Supplementary materials


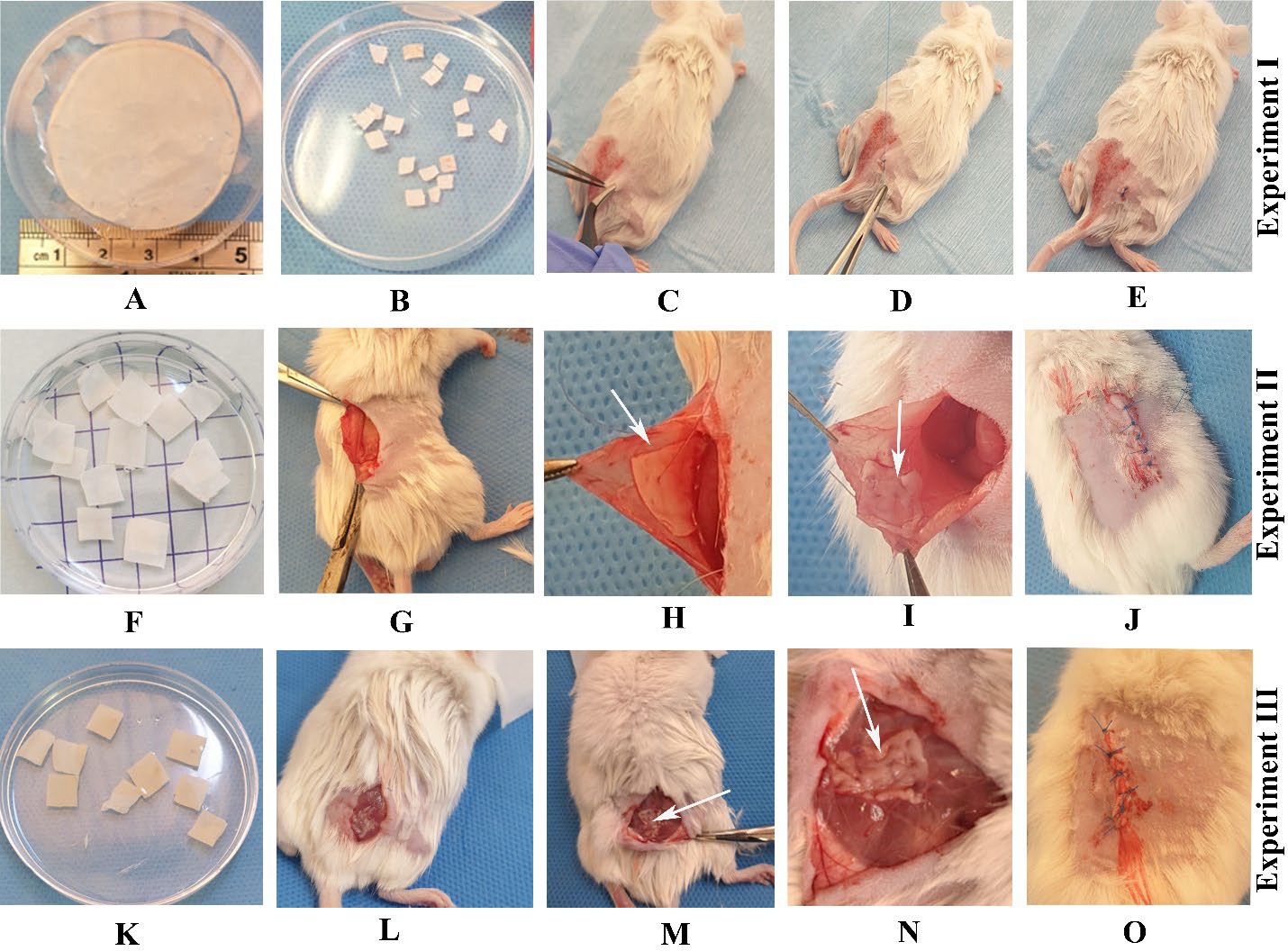


**Supplementary Figure 1.** The surgical procedures on the mice. The morphology of one blood-derived scaffold sample after preparation and washing is shown (A). The dissection of the scaffold is depicted (B). The surgical procedures in Experiment I (subcutaneous; C-E), Experiment II (on the peritoneum; F-J), and Experiment III (on the back muscle; K-O) are demonstrated.


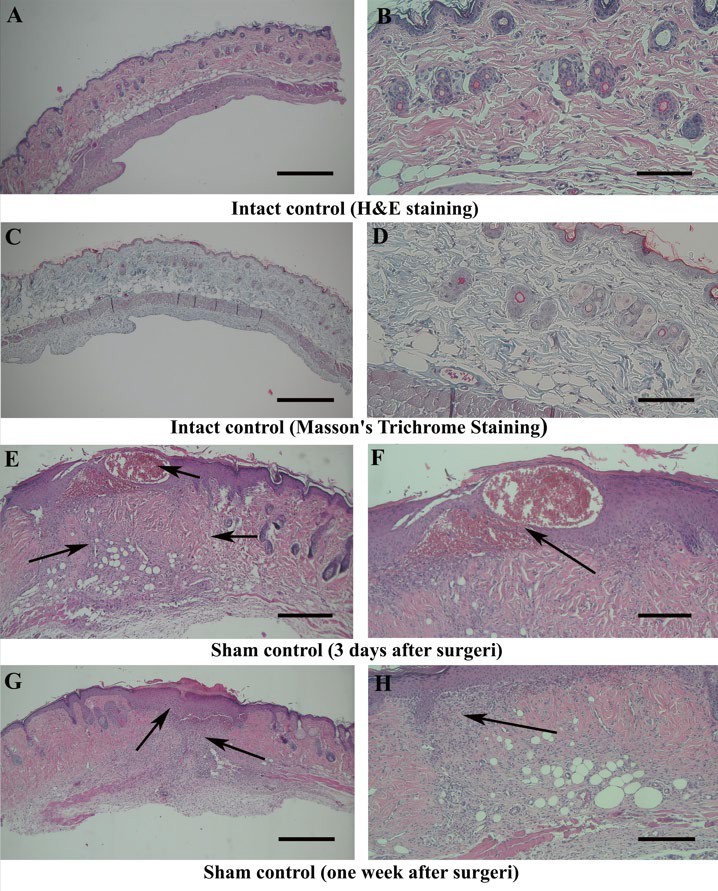


**Supplementary Figure 2.** Normal morphology of mouse skin demonstrated by hematoxylin and eosin staining (A and B) and Masson’s trichrome staining (B and C). Collagen fibers and bundles appear pinkish or green under high-power magnification in the second column. Micrographs of mouse skin in sham-operated samples, taken 3 days and one week after surgery, are presented at both low and high magnifications; black arrows indicate the incision site. Scale bars: A, C; 400 μm, E, G; 200 μm, and B, D, F, H; 50 μm.

**Supplementary Table 1: The Summary of histological changes during 6 weeks of transplantation of human blood-derived scaffold under mouse subcutaneous**.

| **Time** | **Marginal Fibroblast** | **Fibroblast Penetration** | **Scaffold Presence** | **Scaffold Degradation** | **Inflammatory Reaction** | **New Vascularization** | **Marginal Collagen Fibers** |
| --- | --- | --- | --- | --- | --- | --- | --- |
| **Day 3 (N=4)** | +++ | +++ | + | + | +++ | - | + |
|  | + | + | + | ++ | + | - | + |
|  | + | +++ | + | ++ | + | - | + |
|  | ++ | + | + | + | ++ | - | + |
| **Average** | **Moderate, 43.75%** | **Moderate, 50%** | **100%** | **Weak, 37.5%** | **Moderate, 43.75%** | **-** | **100%** |
| **One week (N= 7)** | +++ | +++ | + | +++ | + | - | + |
|  | +++ | +++ | + | + | + | + | + |
|  | ++++ | - | - | ++++ | + | + | + |
|  | ++++ | ++++ | - | ++++ | + | + | + |
|  | ++++ | ++++ | + | ++++ | + | + | + |
|  | ++++ | ++++ | + | ++++ | + | - | + |
|  | ++ | +++ | + | + | + | + | + |
| **Average** | **High, 85.7%** | **High, 75%** | **High, 71.5%** | **High, 75%** | **Weak, 25%** | **High, 71.5%** | **100%** |
| **2 weeks (N=9)** | ++++ | - | + | ++++ | + | - | + |
|  | ++++ | ++ | - | ++++ | + | - | + |
|  | +++ | ++++ | - | ++++ | + | ++++ | - |
|  | - | - | - | ++++ | - | ++++ | + |
|  | ++++* | - | - | ++++ | ++++ | - | - |
|  | ++ | - | - | ++++ | - | - | - |
|  | - | - | - | ++++ | - | - | - |
|  | ++++ | + | + | ++ | +++ | - | + |
|  | ++++ | + | + | ++ | + | ++++ | + |
| **Average** | **High, 69.4%** | **Weak, 25%** | **Weak, 33.33%** | **High, 88.88%** | **Weak, 25.27%** | **Weak, 33.33%** | **Moderate, 55.55%** |
| **3 weeks (N=6)** | - | - | - | ++++ | - | - | - |
|  | - | - | - | ++++ | - | - | - |
|  | - | - | - | ++++ | - | - | - |
|  | +++ | - | - | ++++ | ++ | ++++ | + |
|  | - | - | - | ++++ | - | - | - |
|  | +++ | - | - | ++++ | ++ | - | - |
| **Average** | **25%** | **0** | **0** | **100%** | **16.6%** | **18.3%** | **18.3%** |
| **4 weeks (N=3)** | - | - | - | ++++ | - | - | - |
|  | - | - | - | ++++ | - | - | - |
|  | - | - | - | ++++ | - | - | - |
|  | **0** | **0** | **0** | **100%** | **0** | **0** | **0** |
| **5 weeks (N=2)** |  |  |  |  |  |  |  |
|  | - | - | - | ++++ | - | - | - |
|  | - | - | - | ++++ | - | - | - |
| **Average** | **0** | **0** | **0** | **100%** | **0** | **0** | **0** |
| **6 weeks (N=2)** |  |  |  |  |  |  |  |
|  | - | - | - | ++++ | - | - | - |
|  | - | - | - | ++++ | - | - | - |
| **Average** | **0** | **0** | **0** | **100%** | **0** | **0** | **0** |

* Indicates the presence of some foreign body giant cells

**Supplementary Table 2: The Summary of histological changes during 4 weeks after suturing of human blood-derived scaffold on mouse Peritoneum.**

| **Time** | **Marginal Fibroblast** | **Fibroblast Penetration** | **Scaffold Presence** | **Scaffold Degradation** | **Inflammatory Reaction** | **New Vascularization** | **Collagen Fibers Around Suture** |
| --- | --- | --- | --- | --- | --- | --- | --- |
| **One week (N= 4)** | +++ | +++ | + | ++ | ++ | + | + |
|  | +++ | +++ | + | ++ | ++ | + | ++ |
|  | ++++ | ++ | + | ++ | ++ | + | + |
|  | ++++ | +++ | + | ++ | ++ | + | + |
| **Average** | **High, 85.5%** | **Moderate, 68.75%** | **High, 100%** | **Moderate, 50%** | **Moderate, 50%** | **Weak, 25%** | **Moderate, 31.25%** |
| **2 weeks (N=3)** | ++++ | +++ | + | +++ | +++ | ++ | +++ |
|  | +++ | +++ | - | ++++ | ++ | ++ | +++ |
|  | +++ | ++++ | + | +++ | +++ | +++ | +++ |
| **Average** | **High, 83.33%** | **High, 83.33 %** | **Moderate, 66.66%** | **High, 83.33%** | **Moderate, 66.66%** | **Moderate, 58.33%** | **Moderate, 75%** |
| **3 weeks**  **(N=3)** | ++++ | + | + | +++ | +++ | +++ | ++++ |
|  | ++++ | - | - | ++++ | ++ | +++ | +++ |
|  | +++ | - | - | ++++ | +++ | ++ | ++++ |
| **Average** | **High, 91.66%** | **Weak, 8.33%** | **Moderate, 33.33%** | **High, 91.66%** | **Moderate, 66.66%** | **Moderate, 66.66%** | **High, 91.66%** |
| **4 weeks (N=2)** | +++ | - | - | ++++ | ++ | ++ | ++ |
|  | +++ | - | - | ++++ | + | + | ++ |
| **Average** | **Moderate, 75%** | **0** | **0** | **High, 100%** | **Moderate, 37.5%** | **Moderate, 37.5%** | **Moderate, 50%** |

**Supplementary Table 3: The Summary of histological changes during 4 weeks of transplantation of human blood-derived scaffold on muscle**.

| **Time** | **Marginal Fibroblast** | **Fibroblast Penetration** | **Scaffold Presence** | **Scaffold Degradation** | **Inflammatory Reaction** | **New Vascularization** | **Collagen Fibers Around Suture** |
| --- | --- | --- | --- | --- | --- | --- | --- |
| **One week (N= 4)** | ++ | +++ | + | ++ | ++ | + | + |
|  | ++ | +++ | + | ++ | ++ | + | ++ |
|  | +++ | + | + | ++ | ++ | + | + |
|  | +++ | +++ | + | ++ | ++ | + | + |
| **Average** | **Moderate, 62.5%** | **Moderate, 62.5%** | **High, 100%** | **Moderate, 50%** | **Moderate, 50%** | **Weak, 25%** | **Moderate,%** |
| **2 weeks (N=3)** | +++ | ++ | + | +++ | +++ | ++ | ++ |
|  | ++ | ++ | - | ++++ | ++ | ++ | +++ |
|  | +++ | ++++ | + | +++ | +++ | +++ | ++ |
| **Average** | **Moderate, 66.66%** | **Moderate, 66.66%** | **Weak, 16.66%** | **High, 83.33%** | **Moderate, 66.66%** | **Moderate, 58.33%** | **Moderate, 58.33%** |
| **3 weeks (N=3)** | +++ | + | + | +++ | +++ | +++ | +++ |
|  | +++ | - | - | ++++ | ++ | ++ | ++ |
|  | +++ | - | - | ++++ | +++ | ++ | ++++ |
| **Average** | **Moderate,75%** | **Weak, 8.33%** | **Moderate, 33.33%** | **High, 91.66%** | **Moderate, 66.66%** | **Moderate, 58.33%** | **Moderate, 75%** |
| **4 weeks (N=2)** | ++ | - | - | ++++ | ++ | +++ | ++ |
|  | +++ | - | - | ++++ | + | ++ | ++ |
| **Average** | **Moderate, 62.5%** | **0** | **0** | **100%** | **Moderate, 37.5%** | **Moderate, 62.5%** | **Moderate, 50%** |
